# Participants’ perspectives of being recruited into a randomised trial of a weight loss intervention before colorectal cancer surgery: a qualitative interview study

**DOI:** 10.1186/s12885-024-12464-7

**Published:** 2024-07-05

**Authors:** Amelia Talbot, Susan A Jebb, Claire Foster, Alba X Realpe, Pete Wheatstone, Simon Buczacki, Dimitrios A Koutoukidis

**Affiliations:** 1 Nuffield Department of Primary Health Care Sciences, University of Oxford, Radcliffe Primary Care Building, Radcliffe Observatory Quarter, Woodstock Road, Oxford, OX2 6GG UK; 2 Centre for Psychosocial Research in Cancer, Faculty of Health Sciences, University of Southampton, Southampton, SO17 1BJ UK; 3 NIHR Bristol Biomedical Research Centre, Department of Population Health, University Hospitals Bristol and Weston NHS Foundation Trust, University of Bristol, Canynge Hall. 39 Whatley Road, Bristol, Bs8 2PS UK; 4 Patient and Public Involvement Member, Leeds, UK; 5 Nuffield Department of Surgical Sciences, University of Oxford, Old Road Campus Research Building, Roosevelt Drive, Oxford, UK

**Keywords:** Colorectal Cancer, Interviews, Obesity, Qualitative evaluation, Qualitative research, Weight loss

## Abstract

**Background:**

The period between cancer diagnosis and surgery presents an opportunity for trials to assess the feasibility of behaviour change interventions. However, this can be a worrying time for patients and may hinder recruitment. We describe the perspectives of patients with excess weight awaiting colorectal cancer surgery about their recruitment into a randomised trial of a prehabilitation weight loss intervention.

**Methods:**

We interviewed the first 26 participants from the 8 recruitment sites across England in the ‘CARE’ feasibility trial. Participants were randomised into either usual care (*n* = 13) or a low-energy nutritionally-replete total diet replacement programme with weekly remote behavioural support by a dietitian (*n* = 13). The semi-structured interviews occurred shortly after recruitment and the questions focused on participants’ recollections of being recruited into the trial. We analysed data rapidly and then used a mind-mapping technique to develop descriptive themes. Themes were agreed by all co-authors, including a person with lived-experience of colorectal surgery.

**Results:**

Participants had a mean body mass index (± SD) of 38 kg/m^2^ (± 6), age of 50 years (± 12), and 42% were female. People who participated in the trial were motivated by the offer of structured weight loss support that could potentially help them improve their surgical outcomes. However, participants also had concerns around the potential unpalatability of the intervention diet and side effects. Positive attitudes of clinicians towards the trial facilitated recruitment but participants were disappointed when they were randomised to usual care due to clinical teams’ overemphasis on the benefits of losing weight.

**Conclusions:**

Patients were motivated to take part by the prospect of improved surgical outcomes. However, the strong preference to be allocated to the intervention suggests that balanced communication of equipoise is crucial to minimise disappointment from randomisation to usual care and differential dropout from the trial.

**Clinical trial registration:**

ISRCTN39207707, Registration date 13/03/2023.

**Supplementary Information:**

The online version contains supplementary material available at 10.1186/s12885-024-12464-7.

## Introduction

Randomised controlled trials (RCTs) are the gold-standard for evaluating behavioural interventions; however, almost half of trials fail to recruit their originally specified target sample size [1]. Factors that can reduce recruitment include ineffective communication of trial information, high-time commitments, family influence on the potential participant’s decision, risk of unintended harm, and perceived lack of benefits [2]. Insufficient recruitment can increase the length and cost of RCTs, reduce statistical power, and raise ethical concerns for consenting patients anticipating study benefits [3, 4]. As such, improving recruitment has been identified as an important priority for trials [5].

The Qualitative research Integrated into Trials (QuinteT) recruitment intervention has been developed to support recruitment to RCTs [6, 7]. The QuinteT intervention aims to understand and optimise the recruitment process as it happens using qualitative research methods. These methods include exploring the lived experience of those participating in trials [5, 8]. The lived experience perspective can offer insights into potential participants’ views of the process and progress of an RCT and its treatment arms, and how information provision may influence patient decisions to participate and/or continue to participate in a trial. Findings may provide evidence to intervene in the RCT if necessary [9]. The QuinteT intervention has recently supported five RCTs in surgery/oncology to recruit to target [10].

We conducted a qualitative interview study to explore the lived experience of patients enrolled in the ‘CARE’ trial, inspired by QuinteT intervention [11]. CARE aims to assess the feasibility of preoperative intentional weight loss through a low-energy total diet replacement programme in patients awaiting curative colorectal cancer surgery (ISRCTN39207707, 13/03/2023). Participants were randomised into usual care (the control) or the intervention. The intervention provided participants with a nutritional complete package of formula products and asked them to use four of them per day as the sole source of nutrition until their surgery. The products provided approximately 800 kcal/day and 76 g protein/day and participants could select from 10 flavours of soups, shakes, or porridge [12]. Participants in the intervention also received weekly remote dietetic behavioural support.

We anticipated challenges to recruitment due to clinical teams (the recruiters) potential scepticism about the success of rapid weight loss diets, lack of confidence in weight loss as a treatment, concerns about adherence, and perception that discussing obesity could be stigmatising [13, 14]. Patients may have been hesitant to participate due to an understanding that weight loss is can be a symptom of colorectal cancer rather than an established treatment, and receiving a cancer diagnosis can be a worrying time and trials may be perceived as creating an additional burden [15]. We aimed to explore patients’ motivations to participate and concerns that could hinder recruitment and retention to the CARE trial to inform subsequent recruitment to the trial and recruiters training needs, a planned RCT, and similar trials.

## Methods

The CARE trial received ethical approval from the South Central – Oxford B Research Ethics Committee (Ref: 22/SC/0465).

### Sampling and recruitment

Participants were recruited from eight NHS Trusts in the following areas of England: Cambridgeshire (Cambridge, Peterborough), Derbyshire, Oxfordshire, Devon, South Yorkshire, Dorset, and Worcestershire. Members of the clinical team introduced the trial to potentially eligible participants when they were informed about the potential cancer diagnosis and the likelihood of surgery. Participants were then provided with a detailed information sheet and were made aware that they would be contacted for a telephone interview about their experiences with recruitment and participation in the trial. All participants provided informed consent.

Participants had a BMI ≥ 28 kg/m^2^ (or BMI ≥ 25 kg/m^2^ for people of Black, Asian, or minority ethnic origin [16]) and were listed for curative colorectal resection for cancer with a minimum of 20 days until their scheduled surgery. The trial protocol details the full inclusion/exclusion criteria [11].

### Data collection

AT (female, medical sociologist) and DAK (male, dietitian, chief investigator) conducted semi-structured phone interviews with the first twenty-six participants enrolled in the trial (*n* = 15 by AT, *n* = 11 by DAK). The interviewers had no other relationship with the participants and there was no patient-contact during the trial and had QuinteT training. None of the approached participants declined an interview. We aimed to interview participants as soon as possible after recruitment and ideally within four days from randomisation to minimise recall bias (mean ± SD: 4 ± 2 days, range 1–8). Interviews were conducted using Microsoft Teams [17] between April and October 2023 and lasted, on average, 16 ± 6.5 min (range: 5–31 min).

Interviews started with a narrative question [18], prompting participants to provide a detailed recollection of their experiences of being recruited into this trial (*can you start by walking me through what you remember about being recruited into this trial? )*. This approach helped to map the recruitment process across each site, identify deviations from the protocol, and pinpoint areas of recruitment that may need improving. Subsequent questions explored the participant’s responses to the narrative question and were related to motivations for participating, concerns, and the randomisation process (Supplementary Information 1). The interview guide was based on the team’s experience and existing literature [19–21]. AT and DAK discussed field notes and initial findings after every few interviews.

Interviews were audio-recorded and transcribed verbatim using the artificial intelligence feature on Microsoft Teams. Sections of transcripts used for analysis were checked for accuracy by AT. Transcripts were not returned to participants.

### Analysis

The analysis was led by AT using an inductive-deductive approach, wherein the research questions guided topics for analysis whilst remaining receptive to emerging areas of exploration prompted by the interview data. AT used the rapid analysis sheet [22] method developed by the ‘Rapid Research Evaluation and Appraisal Lab’. This involved creating a table where inductive-deductive topics were listed in the leftmost column, followed by notes from each interview in the subsequent column, and important quotes in the final column. The rapid sheets helped identify gaps in analysis and data saturation, indicating when each inductive-deductive topic returned no additional information.

The rapid method was chosen to quickly and iteratively assess the data to make changes in real time to recruitment processes [23]. This was important because a key factor in the success of the CARE trial was recruiting eligible participants and completing recruitment on time.

The rapid sheet was discussed iteratively with DAK, with further input from AXR (female, psychologist) and CF (female, professor of psychosocial oncology). Once the rapid sheet was complete, AT used the ‘One Sheet of Paper Method’ (OSOP) [24] to mind-map all the issues on the rapid sheet to identify patterns and create descriptive themes. A descriptive thematic approach was chosen to align with our aim to understand how people experience recruitment rather than to generate theory [25]. Co-authors, including a person with lived experience of colorectal surgery (PW), provided feedback on developed themes over email and in team meetings. All co-authors agreed on the final themes. Findings from the rapid and thematic analysis were regularly disseminated to clinical teams as part of the QuinteT intervention.

AT adopted a critical realist ontology, recognising that the identified themes may reflect people’s experiences but are inevitably influenced by her and the rest of the research team’s positionality.

## Results

The first 26 participants enrolled in the trial were interviewed. Their demographics are shown in Table 1. At least one participant from each recruiting site were interviewed except one who had not recruited participants at the time. Most participants lived in a more socioeconomically advantaged area than the UK average based on the Index of Multiple Deprivation [26].

**Table 1:** Participant demographics (*N* = 26)

Participant Demographics	*N* (%)
**Ethnicity**	
Asian White	1 (4) 25 (96)
**Sex**	
Female Male	11 (42) 15 (58)
**IMD quintiles**	
1–2 (least advantaged) 3–4 5–6 7–8 9–10 (most advantaged)	4 (15) 2 (8) 5 (19) 9 (35) 6 (23)
**Randomisation Arm**	
Usual care Intervention	13 (50) 13 (50)
**Recruitment Area**	
Oxfordshire Devon Peterborough, Cambridgeshire Cambridge, Cambridgeshire Derbyshire South Yorkshire Worcestershire Dorset	7 (27) 4 (15) 4 (15) 3 (12) 3 (12) 3 (12) 1 (4) 1 (4)
**Age, years**	**Mean, standard deviation (range)** 50 ± 12 (47–77)
**BMI, kg/m** ^**2**^	**Mean, standard deviation (range)** 38 ± 6 (28–49)
**Weight, kg**	**Mean, standard deviation (range)** 103 ± 19 (72–165)

Three themes were developed, which were observed across both the intervention and usual care group and across participant demographics. Themes with illustrative quotes are presented below. Additional supporting quotes are provided in Tables 2, 3 and 4.

**Table 2:** Additional Supporting Quotes for Descriptive Theme One

Motivations to take part in this trial.	
Note (Code)	Supporting Quote
Motivated to participate due to the potential to lose weight to improve surgical outcomes.	“*The more weight you lose, surely there’s a better chance you have with surgery… You know, obviously doing the liquid diet, surely you must lose weight before the operation.”* (male, 59 years, control)
Motivated to participate due to receiving message about the trial and importance of weight loss to improve surgical outcomes from a perceived credible source.	“*My consultant said I would need he would like me to lose some weight prior to surgery. He then mentioned that there was a trial of this new diet going on in the hospital and would I be interested in hearing from them. So they gave me a few details* [about the trial] *there and then*.” (male, 58 years, intervention)
Motivated to participate as the dietary intervention is scientifically proven to support weight loss and be nutritionally replete.	“*I was quite disappointed because, I was quite keen to do it, but I did understand why* [I was randomised to usual care]*… It was like I said before actually because I’ve been finding it quite difficult to find foods that are low fibre and got enough nutrition. And I knew that this* [dietary intervention] *would have the amount that I needed.”* (female, 59 years, control)
Motivated to participate as the dietary intervention provides structure and an ability to regain control during a time of anxiety associated with a cancer diagnosis and surgery.	“*I was quite looking forward to it actually, because it will take some of the hassle out of life. It might not be a very pleasant diet to be on, but it’s not forever. And it just makes life very, very simple*.” (female, 66 years, control)
Motivated to participate as the dietary intervention includes structured dietitian support.	“*I will be monitored each week with my call with the dietitian. So, I feel like it’s all like safely done to make sure that it’s not going to create a problem for me*.” (male, 71 years, intervention) “*I was interested in it not only for the sake of helping other people in the future, but for the fact that I might actually be able to see a dietitian*.” (female, 78 years, control)
Motivated to participate to contribute to science.	“*They* [study team] *phoned me up about it to see if I’d be interested. But you know, I’m really happy to help anything out*.” (female, 63 years, intervention)

**Table 3:** Additional Supporting Quotes for Descriptive Theme Two

Reservations about the intervention.	
Note (Code)	Supporting Quote
Reservations about feeling full for only a short period of time.	“*It’s quite a shock to the system to suddenly go on a liquid diet of 800 calories a day when you’re used to eating 2000 calories a day of solid food. So that in that in itself might be an issue for some people and until you actually try it, you don’t know*.” (female, 66 years, control) “*If you go hungry, that that could be a problem. But apparently these drinks have got something that makes you feel full… Problem I could see with this kind of diet is being hungry*.” (male, 59 years, control)
Reservations about side effects (e.g., dizziness, constipation).	“*I have spoken to the staff at the gym so they know I have cancer and they know I’m on the shake and soup diet so that if I do have a dizzy turn in there then they’re aware of what what’s caused it and will react accordingly*.” (male, 62 years, intervention) “*I didn’t want to sort of worry about whether I would get constipated with this food* [dietary intervention].” (male, 76 years, intervention)
Reservations about unpalatability of the diet.	“*The fact that you get so many packs of drink, of food and drink because you get one of each, one box of each* [flavour]. *And it did just strike me that there were two flavours that I just couldn’t eat at all*.” (male, 62 years, intervention)

**Table 4:** Additional Supporting Quotes for Descriptive Theme Three

Disappointment when randomised into usual care.	
Note (Code)	Supporting Quote
Disappointment when randomised into usual care due to missing the opportunity to receive structured weight-loss support.	“*I’d been quite disappointed if I hadn’t been in it* [intervention group] *actually because I’d built myself up to the fact it would be good thing to lose some weight and to try and get a bit fitter before my operation*.” (male, 62 years, intervention)
Disappointment when randomised into the usual care due to clinicians’ overemphasis on the benefits of weight-loss.	“*Because I have been told* [by a surgeon] *to try and lose as much weight as possible before my operation. So, I’m not much of a control really, because I’m going to try and lose weight anyway by whatever means*.” (female, 66 years, control)
Nurses showed disappointment when participant randomised into usual care.	“*They* [research nurses] *did pick up my motivation and my willingness to do the diet. They were obviously like “ah no, you’re going for it and everything else” That that’s the way it is.*” (male, 48 years, control) “*She* [research nurse] *clicked a few buttons and said, “oh I’m sorry, but it’s come up that you’re not, you’re in A* [usual care] *and not B.* [intervention]*”* (male, 77 years, control)
Decision to try to lose weight regardless of randomisation into usual care.	*“I probably would have joined* [dietary support service] *again and gone and done it that way* [if not in the intervention]” (male, 58 years, intervention) “*Even though I haven’t gone on to the radical 800 calories a day, I can at least try something to make myself feel better in myself and also hopefully have a good outcome from the operation*.” (female, 72 years, control)

### Theme one: motivations to take part in this trial

Some participants were motivated to join the trial solely to lose weight, while others were solely motivated to potentially improve surgical outcomes and viewed weight loss as a means to achieve this. However, most were motivated by the combined offer of structured support to lose weight to potentially improve surgical outcomes. Some participants mentioned their awareness of the need to lose weight was not prompted by the trial. These participants had already experienced multiple unsuccessful attempts at weight loss, and being invited to the trial was seen as an opportunity to potentially lose weight quickly to improve their recovery from surgery:

“*I am a world champion ‘yo-yo dieter’. My weight will go up and down up and down all my life… So yes, getting the weight down and getting it down quickly is the advantage to me. I perceive that as aiding my recovery at the end*.” (male, 62 years, intervention).

What was motivating for participants was that the dietary intervention had been endorsed by a perceived credible source. For example, one participant described how a message from her surgeon about how losing weight could contribute to a more effective and expedited healing process as a motivator to participate:

“*He* [surgeon] *said, would I take part? Because he thought it might be better in the long run* [to lose weight] *for people who had surgery to get better quicker. You know, if they’ve lost a bit of weight, he felt it would be better. They’d probably heal better and get better quicker. So, I said, yeah, I’d like to take part.*” (female, 73 years, intervention).

The opportunity to receive structured support from a dietitian was also motivating for participants. One participant said, “*The thought of being able to see a dietitian drove me to say, yes, I’ll do it*” (female, 78 years, control). A couple of participants agreed with this motivation, sharing that they had encountered difficulties obtaining dietitian support through the NHS and saw the trial as a way to access these services.

In addition to the surgeon endorsement and dietitian support, most participants said the scientific design of the dietary intervention set it apart from their previous dietary attempts:

“*This* [dietary intervention] *is likely to produce much better results* [than self-management of diet] *because it’s a more scientific, rigid approach, and the sachets all contain all the nutrients*.” (male, 66 years, intervention).

Some participants also described feeling motivated due to a view that the dietary intervention could offer a structured and scientifically informed approach to weight loss during a time of anxiety associated with their cancer diagnosis and surgery:

“*I have got so much on my plate at the moment with what is going on with the diagnosis… So, to have someone put it on a plate for you takes the planning out of the meal planning; all of that goes away… It would have made life a lot easier for me to have been one of the people on the program rather than in the control*.” (female, 66 years, control).

Additional motivations for participating in the trial included contributing to science, supporting the study team, and helping future patients with obesity undergoing the same surgery:

“*One of the reasons for doing it* [the trial] *is because without people trying these things, we’re never going to learn more. And you know, if it helps other people in the future, then that will surely be a good thing.”* (48 years, male, control).

### Theme two: reservations about this dietary intervention

Most participants had reservations about the dietary intervention, with the main one being the significant reduction of energy intake to 800 kcal/day. One participant (male, 62 years, intervention) perceived that having 800 kcal/day would be “*pretty vicious*”, and another (female, 66 years, control) said it would be “*quite a shock*” to go from 2,000 to 800 kcal/day. Participants expressed concerns about potentially being full for only a short time, which was related to negative experiences with previous dietary products:

“*I must say, I was worried about the food because many years ago, I had* [brand of meal replacements], *and that was disgusting, and I just felt hungry all the time.*” (female, 62 years intervention).

The other most noted reservation was the possibility of experiencing side effects due to this reduction in calories. For example, some participants and their families expressed concerns about how the low-energy content of the diet might result in tiredness and affect their ability to carry out everyday activities:

“*She* [partner] *was quite concerned about the study because she had seen on the news that weight loss programmes with a restricted-calorie diet were impacting people’s everyday lives. She was concerned that it would be quite difficult for me with the work that I do and just what I normally do.”* (male, 55 years, control).

The possibility of experiencing side effects as result of the reduction in energy intake was of particular concern to participants who lived with young children. One participant had reservations about whether this reduction would cause dizziness and fainting and the potential impact of this on his ability to play with his six-year-old son:

“*My son will want to go out and use bike and he’ll want me to race him on the scooter or on the bike against him and I’ll burn energy there. And it’s just that balance of what happens if you go too far, is there a risk of you fainting?*” (male, 62 years, intervention).

Some participants also had reservations about whether the reduction in energy intake would impact their mood. One participant, who had already started the diet two days prior to the interview, said his wife had noticed his sudden irritability:

“*800 calories a day is going to possibly cause issues with temper, and my wife has already mentioned my temper to me. Hopefully* [I am] *not going to turn grumpy by next week*.” (male, 62 years, intervention).

A small number of participants had reservations about the possibility of constipation, a common side-effect of the intervention:

“*I think the main struggle was worry was that I don’t tend to get constipated quite a lot, and I wondered whether that* [dietary intervention] *would make it worse*.” (female, 76 years, intervention).

Additional reservations were raised about the potential unpalatability of the diet, with a couple of participants specifically mentioning that it might discourage them or lead to withdrawal from the trial:

“[I was worried about] *whether they* [meal replacement products] *would taste okay because I didn’t want to be putting myself through five weeks of something that didn’t taste reasonable*.” (male, 58 years, intervention.

### Theme three: disappointment when randomised into usual care

Most participants randomised into usual care described disappointment due to missing the opportunity for structured weight-loss support.

“*We* [participant and his wife] *thought it would be nice if we were chosen to go onto the sachets and the soups to see if that could allow me to lose a bit more weight before the operation… So yes, it was a little bit disappointing*” (male, 75 years, control).

Participants in the intervention also described feeling pleased that they had not missed out on the dietary intervention. One participant said, “*Luckily, the computer picked me*….” (female, 63 years, intervention).

This disappointment of being randomised into usual care was linked to the clinical teams’ disproportionate emphasis on the benefits of losing weight. One participant understood the need for usual care but described feeling disappointed due to her surgeon’s emphasis on the value of losing as much weight as possible before surgery:

“*While I understand the need for a control and I understand why half of them are on it, and half of them are off it, my own agenda is different to that of the experiment really because I have been told to try and lose as much weight as possible before my operation*.” (female, 66 years, control).

Some participants also noticed the reactions of research nurses upon randomisation into usual care. These participants reported that nurses would apologise and exhibit signs of disappointment when delivering this news, which reinforced their disappointment at their allocation to the usual care group:

“*I was quite disappointed because we went through all of the weigh-in and questions and what have you, and then the nurse pressed the button on the computer, and she went, “Oh no*.” (female, 78 years, control).

Due to this disappointment, one participant described her intention to source the intervention diet (she was unsuccessful in doing so):

“*I’m going to try and lose weight anyway by whatever means. Whether I go to the company… whether I go to them direct and buy the shakes and soups as a private individual, or whether I follow my own diet.”* (female, 66 years, control).

Most participants in the usual care group agreed that they would try to lose as much weight as possible before surgery. Some of these participants recalled asking surgeons about how to lose a significant amount of weight:

“*I was not selected to go on to the restricted diet, but I did ask for dietary advice* [from the surgeon] *because it makes sense to lose a lot of weight before an operation, and that was fully explained to me. I can at least try something to make myself feel better in myself and also hopefully have a good outcome from the operation*.” (female, 72 years, control).

However, most participants acknowledged that they might not achieve as much weight loss through self-management as they would using the structured diet provided in the intervention. A few of these participants were concerned about the potential consequences of not achieving significant weight loss on their surgical outcomes:

“*I was randomised to the non-diet* [usual care] *group… It’s* [self-management of diet] *not going to help me deal with my weight any sooner than what you’re doing at the moment* [dietary intervention]. *So, I’m not going to lose weight as quickly as I would like, and perhaps that might not be beneficial for the surgery. But, it is what it is, what it is.”* (male, 55 years, control).

## Discussion

Our qualitative study shows that patients with excess weight awaiting colorectal cancer surgery reported wanting to take part in the CARE trial because they were motivated to engage in structured weight loss support that could potentially help them improve their surgical outcomes, and that the research would help others. While the positive attitudes of clinicians towards the trial facilitated recruitment, it also contributed to disappointment when participants were randomised to usual care.

However, many participants were unsure on whether they would find the dietary programme appealing and early data from our screening logs suggests many patients declined the trial because they perceived the specific weight loss programme to be unacceptable. Reservations about the potential unpalatability of unfamiliar dietary products and potential hunger are common among participants in total diet replacement programmes [27, 28]. We used these findings to provide feedback to recruiters around discussing concerns on palatability and potential side effects. We specifically asked recruiters to use the phrase “give it a go” based on work indicating its effectiveness in prompting participant engagement with weight-loss behaviours [29, 30]. Recruiters were also advised to inform participants about the free provision of meal replacement products and weekly appointments with a dietitian [31], who would support them on managing potential side effects and issues with palatability. A wider choice of dietary products or food-based weight-loss interventions may have improved acceptability and should be considered if the trial shows weight-loss to improve treatment outcomes.

No participant mentioned being interested in joining due to perceiving their cancer as a “teachable moment”. This result was surprising as previous studies have proposed that the time of cancer diagnosis can serve as a teachable moment for prompting lifestyle changes [32, 33]. However, this finding does align with one study which found that a prostate cancer diagnosis in patients with obesity was not followed by weight loss [34]. It was more that patients saw this as a moment to get the support they described needing to be able to manage their weight. Moreover, the specific attributes of the dietary intervention, such as the prospect of significant weight loss to potentially reduce the risk of post-operative complications, clinician endorsement, dietitian support, optimal nutritional composition, and provision of structure during an uncertain time were perceived to play an important role in motivating participants toward weight loss. Qualitative studies have also found that most of these features contribute to motivation for weight loss and adherence to total diet replacement programmes [19, 35].

A previous systematic review [15] suggested that the anxiety that can accompany a cancer diagnosis and impending surgery might deter some people from participating. Data from people declining our trial will be reported separately, but some of those who agreed to take part reframed this expectation and described feeling that the structured support of this dietary intervention could help them control their anxiety. Earlier research has also highlighted that the potential side effects of this diet, such as tiredness, fatigue, irritability, diarrhoea, and constipation can act as deterrents to participation in pre-rehabilitation services [36, 37], which were concerns here. At the same time, some studies have shown that pre-rehabilitation can alleviate some of these symptoms [38]. We suggest that information from a credible source emphasising the benefits of the dietary intervention could encourage participation and provide space for participants to weigh up potential disadvantages.

Most participants were disappointed when they were randomised into usual care which they perceived as a missed opportunity to benefit as a consequence of the clinical teams’ disproportionate emphasis on the benefits of losing weight. In addition, some participants described how the emphasis on losing weight encouraged them to pursue weight loss regardless of their randomisation into usual care. If participants are successful with losing weight or sourcing the intervention diet, this could potentially impact the weight change between study arms. Our findings support previous qualitative studies where health professionals have described difficulty with conveying equipoise due to their perception of the potential advantages of the treatment being offered [39]. For example, in one interview study with recruiters across six RCTs with poor recruitment, some doctors experienced discomfort and emotion with conveying equipoise due to their clinical instincts and concerns about patient eligibility and safety [40].

This lack of perceived equipoise is problematic for trials because of the link to ‘conditional altruism’. This has been described as the willingness to contribute to science as an initial motivator, but one that is unlikely to lead to trial participation or continuation unless people also recognise that participation will benefit them personally [41]. This can be a threat to the success of a trial if it leads to differential follow-up in the two groups. For the CARE trial, the risk for this is low, since the outcomes will be measured based on a follow-up appointment in routine care. Nonetheless, as part of the QuinteT intervention, we consistently emphasised to recruiters the need to balance their communication of the potential benefits of the diet intervention with the inherent uncertainty surrounding treatment superiority, and to complement this with a clear explanation of the value of randomisation to the integrity and value of the trial. Additionally, we advised against using gambling-related metaphors like ‘toss of a coin’ and computer-agency descriptions like ‘the computer chooses’ as an observational study showed they can impede recruitment [40].

We proposed to collect audio-recordings of recruitment conversations in addition to interviews to reduce recall bias and explore the interactional dynamics of recruitment conversations [10, 42]. However, we were unsuccessful in encouraging most recruiters to record these conversations and the returned recordings did not provide additional valuable information. Our experience resonates with a survey [43], which showed that clinicians can perceive consultation recordings as a potential threat, inhibiting their ability to provide optimal advice. However, we recognise the value of deep conversational analysis of such recordings to identify communication strategies to most effectively convey equipoise in RCTs.

The study has strengths and limitations. We interviewed the first 26 randomised participants from across all recruitment sites aiding the transferability of the findings. We believe data were saturated as no new information was elicited from the last interviews. The findings were also developed with an interdisciplinary team and a person with lived-experience of colorectal surgery. Limitations include that we did not interview patients who declined to participate in the trial. The majority of participants were of White ethnicity, but this is in line with the demographic of participants with colorectal cancer in England [44]. Although we told participants we welcomed positive and negative feedback, and the interviewers did not have a relationship with the participants, the possibility for social desirability remains [45]. We interviewed most participants as soon as practically possible after randomisation, but there may have been some recall bias [45].

## Conclusions

In conclusion, patients with excess weight awaiting colorectal cancer surgery decided to join a randomised trial testing a structured weight loss intervention because they were motivated to lose weight to help them improve their surgical outcomes. Positive attitudes of clinicians towards the dietary intervention facilitated recruitment but the overemphasis on the benefits of weight loss increased the feeling of disappointment among participants randomised to usual care.

### Electronic supplementary material

Below is the link to the electronic supplementary material.

Supplementary Material 1

## Data Availability

The data that support the findings of this study are available on request from the corresponding author [DAK]. The data are not publicly available as they contain information that compromises the privacy of research participants.

## Abbreviations

RCTs: Randomised controlled trials
