# Supplementary material for: Participants’ perspectives of being recruited into a randomised trial of a weight loss intervention before colorectal cancer surgery: a qualitative interview study
Source: BMC Cancer. 2024 Jul 5;24:802. doi: 10.1186/s12885-024-12464-7 (PMC11225294; doi:10.1186/s12885-024-12464-7)
Supplement: Supplementary file 1 — Supplementary Material 1 [file 12885_2024_12464_MOESM1_ESM.docx]

**SUPPLEMENTARY FILE ONE: GUIDE FOR THE QUINTET QUALITATIVE INTERVIEW WITH PARTICIPANTS POST-RANDOMISATION**

**Topic Question Prompt**

| Introduction Thank you for taking the time for the interview. The aim of the interview is to tell me your thoughts about how you found the study processes so far. We welcome all comments, positive and negative and indeed sometimes the negatives comments are the most helpful, so please feel free to express your views. Can you start by walking me through your recollections of being recruited into this trial?  When did you first hear about this study?  - From whom?  - What were your initial thoughts? | - Cancer nurse specialist  - Surgeon  - Researcher/research nurse |
| --- | --- |

What do you understand about the study? What is it trying to find

out?

Tell me about how you made the decision to join the study. At what point did you

decide? What/who helped you to decide?

- Improve recovery

- Prepare for surgery

- Clinician endorsement

- Feel in control

- Teachable moment

What has helped you sign up?

Any aspects of the study that you were uncertain or
concerned about?

How did you feel about finding out that the choice of
having or not having the diet was done at random? Did it
make sense to you why it had to be done that way?

How did you feel when you find out that you were
randomised to the [weight loss/control] group?

| In your opinion, what are the possible benefits and risks of the diet?  How have you found the study visit?  Close down Any further comments? Thank you for taking part in the interview. | - Interfering with daily life  - Confidence following it - Acceptability |
| --- | --- |
